# Optimization of Plasmonic Gold Nanoparticle Concentration in Green LED Light Active Dental Photopolymer

**DOI:** 10.3390/polym13020275

**Published:** 2021-01-15

**Authors:** Katalin Bukovinszky, Melinda Szalóki, István Csarnovics, Attila Bonyár, Péter Petrik, Benjámin Kalas, Lajos Daróczi, Sándor Kéki, Sándor Kökényesi, Csaba Hegedűs

**Affiliations:** 1 Department of Biomaterials and Prosthetic Dentistry, Faculty of Dentistry, University of Debrecen, H4032 Debrecen, Hungary; bukovinszki.katalin@dental.unideb.hu (K.B.); szaloki.melinda@dental.unideb.hu (M.S.); 2 Department of Experimental Physics, Institute of Physics, Faculty of Science and Technology, University of Debrecen, H4032 Debrecen, Hungary; csarnovics.istvan@science.unideb.hu; 3 Department of Electronics Technology, Budapest University of Technology and Economics, H1111 Budapest, Hungary; bonyar@ett.bme.hu; 4 Centre for Energy Research, Institute of Technical Physics and Materials Science (MFA), H1121 Budapest, Hungary; petrik.peter@ek-cer.hu (P.P.); kalas.benjamin@ek-cer.hu (B.K.); 5 Department of Solid State Physics, Institute of Physics, Faculty of Science and Technology, University of Debrecen, H4032 Debrecen, Hungary; lajos.daroczi@science.unideb.hu; 6 Department of Applied Chemistry, Institute of Chemistry, Faculty of Science and Technology, University of Debrecen, H4032 Debrecen, Hungary; keki.sandor@science.unideb.hu; 7 Department of Electrical and Electronic Engineering, Institute of Physics, Faculty of Science and Technology, University of Debrecen, H4032 Debrecen, Hungary; kiki@science.unideb.hu

**Keywords:** gold nanoparticles, photopolymerization, surface plasmon resonance, nanoplasmonics, Irgacure 784, dimetacrylate resin

## Abstract

Gold nanoparticles (AuNPs) display surface plasmon resonance (SPR) as a result of their irradiation at a targeted light frequency. SPR also results in heat production that increases the temperature of the surrounding environment, affecting polymerization. The aim was to investigate the SPR effect of AuNPs on a dimethacrylate-based photopolymer system. The tested composites were designed to overlap the illumination required for the polymerization and the plasmon effect. The 5 nm-sized dodecanethiol capped AuNPs were applied in different concentrations in the matrix that were irradiated with green light (λ = 532 nm), where the Irgacure 784 photoinitiator also absorbs the light. The plasmonic effect was investigated for the refractive index change by surface plasmon resonance imaging (SPRi) supplemented by ellipsometry. Moreover, optical transmission and transmission electron micrographs (TEM), diametral tensile stress (DTS), and confocal Raman spectroscopy was performed to determine the degree of conversion (DC) at 1.0, 1.4, and 2.0 mW/cm^2^ light intensities. It was found that the optimal conditions were at 0.0208 wt% AuNPs concentration and 1.4 mW/cm^2^ light intensity at which the refractive index change, DTS, and DC data were all maximal. The study confirmed that AuNPs are applicable to improve the polymerization efficiency of dental composite resin.

## 1. Introduction

The continuous improvement of dental composites is permanently in the focus of manufacturers, dental professionals, and researchers [1]. The characteristics and multiple properties of these materials are determined by those dimethacrylate monomers that are responsible for the formation of its resin matrix. The most commonly used monomers in dental composites are bisphenol A diglycidyl dimethacrylate (Bis-GMA) and its ethoxylated analog (Bis-EMA) and urethane dimethacrylates (UDMA) along with low molecular weight diluents, usually ethylene glycol derivatives, such as triethylene-glycol dimethacrylate (TEGDMA). The effect of chemical composition and different ratios of dimethacrylates (Bis-GMA, UDMA, TEGDMA) on mechanical properties has been demonstrated already in experimental resin composites by Asmussen and Peutzfeldt [2]. The challenge is to create a resin matrix with low polymerization shrinkage and better depth of cure or degree of conversion along with improved mechanical properties, aesthetics, and biocompatibility [3]. As they are photosensitive materials, the successful photocuring process requires an efficient initiator molecule and adequate light energy with compatible wavelength. The frequently used initiator is champhoroquinone (CQ) that can be activated by 400–500 nm (maximum at 470 nm) wavelength blue light. CQ is a solid yellow diketone compound with an unbleachable chromophore group, which leads to an undesirable yellowing effect on the final esthetic appearance of a cured material. Furthermore, CQ needs a reducing agent to generate free radicals to initiate the polymerization of photo-activated resin-based filling materials. The tertiary amine used can also add uneven yellow discoloration to the cured restoration. These disadvantages of CQ motivated the researchers to find alternative initiator molecules. Acylphospine oxide derivates such as diphenyl (2,4,6-trimethylbenzoyl) phosphine oxide (lucirin TPO), phenylbis (2,4,6-trimethylbensoyl) phosphine oxide (BAPO), and the pale yellow liquid 1-phenyl and 1,2 propenedione (PPD) have been suggested as an alternative photoinitiator in dental composites [1,4].

The other crucial requirement of successful photopolymerization is the question of applying effective light sources. From this point of view, the absorption spectrum of the applied photoinitiator should correlate with the spectral emission profiles of the light-curing units (LCU) [5]. Historically, several types of LCU were used in dentistry such as quartz-tungsten-halogen lights, argon-ion lasers, plasma arc lights, and light-emitting diodes (LEDs). LEDs proved to be the most successful thanks to their long life service and, in addition, they are compact, portable, and energy-efficient [6]. As a result of the free radical polymerization of dimethacrylate based dental composite matrix, a three-dimensional connective crosslinked polymer network is formed. The extent of polymerization is quantified by comparing the amount of remaining double bonds in the polymer structure to the initial amount. This ratio, expressed in %, is termed degree of conversion (DC). Generally, DC values vary in a wide range of resin-based dental composite types, from about 35–77% [7]. Achievement of maximum DC of resin materials requires the presence of optimal circumstances. There are various factors, which affect the polymerization process of resin-based composites, such as the composition of the reaction mixture, curing mode, light-curing time, increment thickness, light-curing units used, post-irradiation time, cavity diameter and its location, distance of the light-curing tip from the surface, the substrate used, type of filler, and temperature [1]. Increased filler-matrix ratio leads to a reduced degree of conversion, as filler particles can inhibit the polymerization, and could have an effect on curing-light permeability, too. Monomer composition, initiator concentration, and co-initiator/inhibitor system, also affect the depth of cure and hence the degree of conversion of the resin-based composites [1,8,9,10,11]. As a solution for the decreasing effect of the inorganic fillers on the curing-light permeability of the resin matrix, the rising temperature may be one alternative. The temperature during polymerization can significantly affect the polymerization efficiency, as an increase from room temperature (22 °C) to mouth temperature (35 °C) results in increased DC (6–10%) as reported by several researchers [9,10,11]. 

The application of noble nano metals in dental composite is well-known in the literature. The purpose of these investigations is to create resin based esthetic filling materials to protect the formation of secondary caries along the borderline of the cavity, and adjacent tooth structure. The experimental materials release bactericidal noble metals gold and silver ions to effectively prevent the survival of cariogenic bacteria [12,13].

Another application of noble metal nanoparticles (NPs) enables the necessary increase in temperature and efficiently releases heat under light irradiation. The heat then diffuses away from the NPs and leads to an elevated temperature of the surrounding medium. This opens up a new set of applications in nanotechnology and gives rise to a new promising field of plasmonic heating [14]. 

Gold nanoparticles are highly customizable in size, shape, and surface as well as being biocompatible and chemically stable under various conditions. In addition, they have controllable optical-electronic properties suitable for medical photothermal therapeutic application and biological sensing [15].

Based on this idea, we have selected spherical gold nanoparticles (AuNPs) for this purpose. However, the resonant excitation of gold nanoparticles requires different wavelengths of light than those used in the dental photocuring system (blue light). Consequently, we have constructed a green-emitting LED curing unit, and our device required a photoinitiator sensitive to the wavelength of 532 nm of green light [16]. For this reason, the well-known and widely applied Irgacure 784 photoinitiator was chosen for these studies [17]. 

The aim of our work was to investigate the thermoplasmonic effect of the green LED excited spherical gold nanoparticles on the physical properties of the dimethacrylate-based dental resin.

## 2. Materials and Methods

### 2.1. Initial Materials

The following materials and chemicals were used for sample preparation: bisphenol-A glycol dimethacrylate (Bis-GMA) (Sigma–Aldrich Co., St. Louis, MO, USA), triethylene glycol dimethacrylate (TEGDMA) (Sigma–Aldrich Co., St. Louis, MO, USA), diurethane dimethacrylate (UDMA) (Sigma-Aldrich Chemie GmbH, Steinheim, Germany), photoinitiator, Irgacure 784 (IRG) (BASF Hungary Ltd., Budapest, Hungary), dodecanethiol functionalized 5 nm gold nanoparticles (AuNP) (Nanoprobes Inc., Yaphank, NY, USA). All materials were used without further purification.

### 2.2. Preparation of Polymer Nanocomposites

The photocurable resin matrix containing 0.5 wt% Irgacure 784 as the photoinitiator was a mixture of Bis-GMA, TEGDMA, and UDMA monomers in 21.4:25.4:53.3 weight ratio, respectively. The specimens prepared for different kinds of measurement were stored under the same conditions, at room temperature; 1 mg/mL stock solution was prepared from AuNP in high-performance liquid chromatography (HPLC)-grade toluene (VWR International LLC, Debrecen, Hungary). From this stock solution, different amounts of AuNPs were added to the premeasured UDMA monomer, thus composites at different gold concentrations were obtained (Table 1). AuNPs were dispersed by means of an ultrasonic bath. Toluene was evaporated off in a vacuum (55 °C, 3 mbar) with the help of a vacuum rotating distillation unit (Heidolph Hei-VAP Precision, Heidolph, Schwabach, Germany) until constant weight. The reference dental resin (Ref) (Table 1) as a blind sample was prepared by the same procedure as the samples containing gold particles except that AuNPs were not added to the monomer, only toluene as the solvent. After evaporating toluene, premeasured Bis-GMA, TEGDMA, and photoinitiator were added to UDMA-containing AuNPs and homogenized by an ultrasonic bath and stirred overnight at room temperature.

### 2.3. Investigation of the Progress of Photopolymerization of Gold Nanoparticle (AuNP)-Doped Nanocomposites—Surface Plasmon Resonance Imaging (SPRi) and Ellipsometry Measurements

These measurements helped us to choose the optimal AuNPs concentration for further analysis of physical properties. The photopolymerization process was studied by measuring the change of the refractive index during irradiation. The refractive index change caused by a green LED light source (output P = 1.0 mW/cm^2^, 1.4 mW/cm^2^ and 2.0 mW/cm^2^) was measured by surface plasmon resonance imaging (SPRi). For the measurements, a custom-built SPRi instrument [18] was used, which utilizes Kretschmann optical configuration with a 680 nm superluminescent light source and a 1 MP charge-coupled device (CCD) camera with a 25° range of incident angle. In this configuration, the positions of the light source and the camera are fixed, and only the prism holder platform can be rotated to scan and find the inflection point of the SPR peak to maximize the sensitivity. There are no moving parts during the measurements. Although the SPRi instrument was designed and adjusted to measure primarily in aqueous environments, we re-calibrated the device to be able to measure the much higher refractive indexes of polymers, such as those of the investigated nanocomposites. For SPRi measurements, the investigated nanocomposites were placed onto an SPRi chip (50 nm gold deposited on a glass substrate, purchased from Mivitec, Germany), then a thin layer was formed by pressing an ultraviolet–visible (UV–VIS) transparent polyester film on the top of the nanocomposites. Real-time changes in the refractive index of the media were monitored during the whole illumination. SPRi results (kinetics) are always given as absolute changes in the refractive index of the nanocomposites with time. Due to the nature of the SPRi measurements, only kinetics measured simultaneously on sample-pairs—on the same chip, at the same time—can be compared with each other, which is true for all the kinetics curves presented in our figures. No comparison between the individual measurements (e.g., comparison of kinetic curves from separate measurements) was made. Based on SPRi measurement (causing the largest refractive index change), two nanocomposites were chosen for further investigation. The refractive index, before and after photopolymerization of the Ref, the Au1, and Au2 samples were measured by ellipsometry (Woollam M-2000DI, Lincoln, NB, USA). For the irradiation, the same light source and parameters were used for the SPRi measurements. The obtained data with ellipsometry was used to normalize the SPRi kinetics and to obtain an absolute change in the refractive index. All the samples were irradiated for 180 s. The largest change of the refractive index was obtained after 120–135 s.

In the case of the refractive index measurement for each sample and light intensity, *n* = 5 samples were investigated. In the case of the SPRi measurements for each sample and light intensity, *n* = 3 samples were studied.

### 2.4. Analysis of the Plasmonic Effect and Dispersion of AuNPs in Polymer Nanocomposites—Optical Transmission Measurements and Transmission Electron Microscopy (TEM) Analysis

Based on the SPRi measurements, two AuNP concentrations were chosen for further analysis. The optical transmission measurements helped us to present the plasmonic effect of nanoparticles by light absorption. The TEM records can certify the distribution of AuNPs in the cured matrix. The optical transmission of the reference sample, the nanocomposite containing AuNPs, and AuNPs in toluene were measured by spectrophotometer (Shimadzu UV-3600, Kyoto, Japan), while the spectral irradiance distribution of the light sources were detected with a spectroradiometer (EKO Instruments, LS-100, De Haag, Netherlands). The measured data were normalized to reference sample data. In our experiments, we used an LED light source (Megaled, 3W green power LED, Budapest, Hungary) to polymerize the resin matrix. The LED light source was described in our previous study [16]. The optical spectra of the light source were measured by fiber optical spectrophotometer (Ocean Optics, USB650, Dunedin, FL, USA). It was found that the peak of the LED light source was at 532 nm. The intensity of the light sources was measured by the power meter setup (ThorLabs, PM100, Newton, NJ, USA).

The AuNPs distribution in the polymerized resin was investigated with Transmission Electron Microscopy (TEM, Jeol-2000FX-II, Tokyo, Japan) equipped with a Bruker EDS system. The TEM samples were produced by ultramicrotomy (LKB Ultrotome 4801A Stockholm-Bomma, Sweden) from the cured polymer blocks containing Au NPs. The sections were floated onto copper microgrids. Samples were investigated at 200 kV accelerating voltage.

### 2.5. Investigation of the Physical Properties of the Polymer Nanocomposites—Diametral Tensile Strengths (DTS) and Degree of Conversion Measurements (DC)

The reference resin and resins containing AuNPs were polymerized for 3 min in a Teflon mold covered by a polyester strip at green LED light intensities of 1.0, 1.4, and 2.0 mW/cm^2^. The polymerization was performed in a dark room. The dimension of specimens was 3 mm in height and 6 mm in diameter for mechanical testing. Before mechanical testing and Raman measurements, the specimens were stored at room temperature for 24 h.

Diametral tensile strength (DTS) was measured on polymerized specimens of reference resin (*n* = 10) and AuNPs doped resins (Au1 (*n* = 10) and Au2 (*n* = 10)) with the help of a mechanical testing device (INSTRON 8874, High Wycombe, UK) equipped with 25 kN load cell. The crosshead speed was 1.00 mm/min. The DTS strength data were calculated based on MSZ EN ISO 604:2003 [16].

Characterization of the degree of conversion (DC) was studied with Raman Microscopy at different depths. The degree of conversion of specimens (*n* = 3) (3 mm in height and 6 mm in diameter) was measured by confocal Raman spectroscopy (Horiba LabRam HR Evo, Palaiseau, France). On the sample surface, 6 measurements were performed on different points. During the work, a 633 nm laser was used as an excitation source, and the measurement time for each sample was 20 s, the accumulation was 10. The excitation beam was focused onto the sample surface with a 10× lens, while the 600 line/mm grating was used for the measurement. The intensity of the laser was reduced to <1 mW at the sample surface to avoid damages and light-induced transformation of the samples. Spectra were baseline-corrected with the built-in algorithm of the Raman spectrometer software, then normalized and fitted with a set of Gaussians to obtain the Raman peak parameters. The error of the fitting and calculation of the investigated peaks, their parameters, and the estimation of the degree of conversion was about 0.5–1%. For the Raman spectrum, the analyte was placed on a bare glass slide under the same conditions and in the same amount. The degree of conversion was calculated based on this equation:

$$\mathrm{DC}\;\left(\%\right)=100\;\times \;\left[1-\left(\frac{\frac{I_{2\mathrm{polymerized}}}{I_{1\;\mathrm{polymerized}}}}{\frac{I_{2\;\mathrm{unpolymerized}}}{I_{1\;\mathrm{unpolymerized}}}}\right)\right]$$

where I_1_ and I_2_ correspond to the area under the peaks at 1610 cm^−1^ and 1640 cm^−1^, respectively [19].

### 2.6. Statistical Analysis for Diametral Tensile Stress (DTS) and Degree of Conversion (DC)

Statistical analysis for DTS and DC Data was performed using Student’s t-test with SPSS 17.0 software (IBM, Armonk, NY, USA). All of the tests’ accuracies were set at a significance level of 0.05. The Kolmogorov–Smirnov Test revealed that the data showed normal distribution and variances are equal across groups based on homoscedasticity Bartlett’s Test.

## 3. Results

As outlined in the Introduction, our aim was to improve the mechanical properties of dental resins by applying gold nanoparticles, as nanoplasmonic materials embedded into a resin matrix (due to the heat effect caused by the illumination of AuNPs). The plasmonic effect of the nanoparticles was achieved by green LED light illumination, and the photopolymerization reactions in the presence and absence of AuPNs were monitored.

### 3.1. Surface Plasmon Resonance Imaging (SPRi) and Ellipsometry Measurements

In these measurements, the aim was to choose the optimal AuNPs concentration for further analysis of physical properties.

The samples (Ref, Au1, Au2, Au3, Au4, Au5, and Au6) were illuminated with green LED light of three different intensities (1.0 mW/cm^2^, 1.4 mW/cm^2^, and 2.0 mW/cm^2^) to compare the influence of the light intensity on the photopolymerization kinetics and the mechanical properties. Moreover, the refractive index of the Ref. sample was measured before and after illumination using the same three light intensities (see Table 2). As can be seen from the data of Table 2, higher light intensity results in a higher refractive index change.

**Figure 1:**
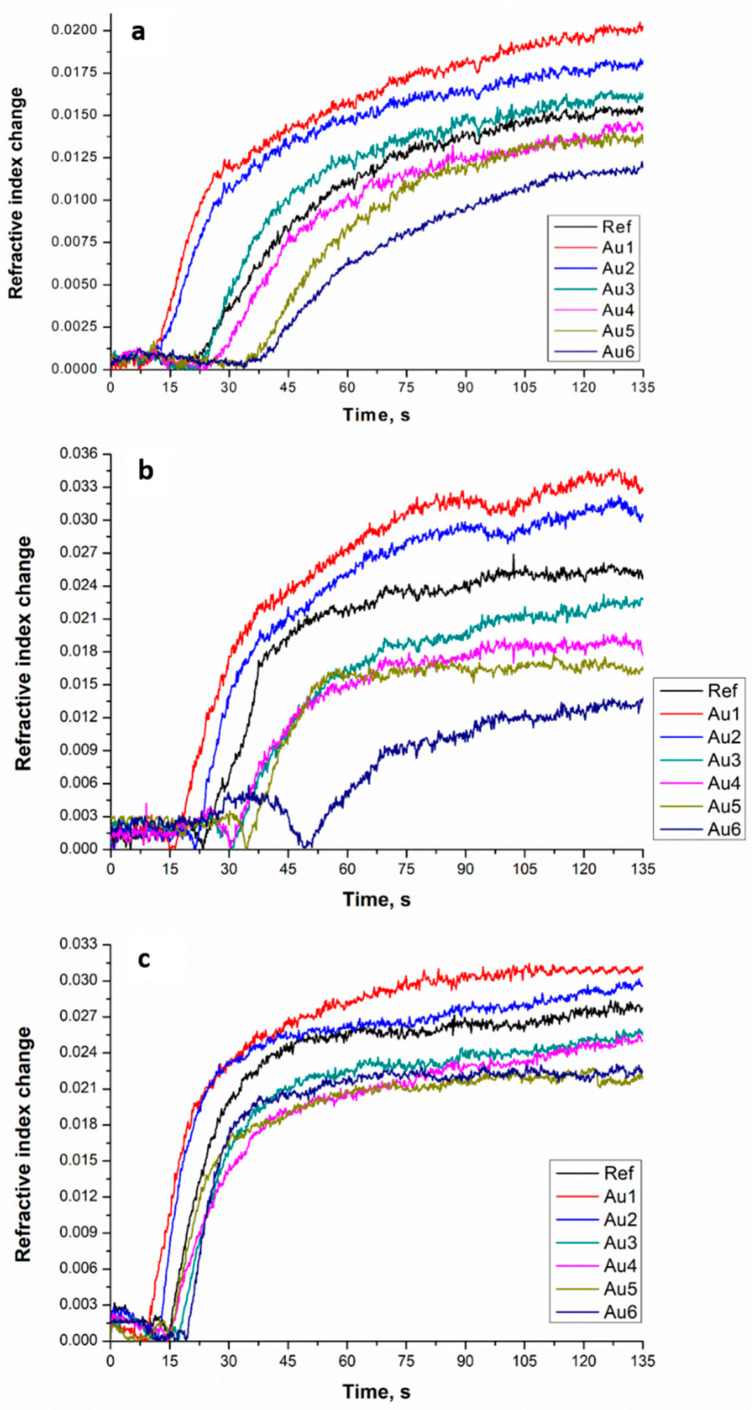
Surface plasmon resonance imaging (SPRi) kinetics of the investigated samples upon irradiation at different light intensities: (**a**) 1.0 mW/cm^2^, (**b**) 1.4 mW/cm^2^ and (**c**) 2.0 mW/cm^2.^

The refractive index change determined by SPRi for the seven samples at three different light intensities were normalized by the values obtained for the Ref sample with ellipsometry (see Figure 1).

It can be seen from Figure 1, that irradiation with a LED light source caused the increase of the refractive index, which can be related to progress of the polymerization processes in nanocomposites. The addition of nanoparticles influences the rate of the photo-polymerization. Indeed, it can also be seen that induction time (time interval before polymerization starts) for the studied samples is different. For samples Au1 and Au2, this time is shorter in comparison to the Ref sample, while for others with AuNPs, it is longer with respect to the reference sample. As for the light intensity at 1.0 mW/cm^2^, the samples Au1, Au2, and Au3 have a larger refractive index change in comparison to the Ref sample, while for other samples, it is lower. For the second intensity, 1.4 mW/cm^2^, only Au1, and Au2 samples have a higher refractive index change than the reference one, others have a lower value than it. At 2.0 mW/cm^2^, the situation is the same as for the previous case, but the difference between the Au1, Au2, and Ref samples is not significant. In comparison, other samples containing a higher amount of gold nanoparticles showed a lower refractive index change.

The rate of polymerization for the samples Au1 and Au2 at the three intensities were different (see Table 3): the highest rate was found at 1.4 mW/cm^2^, a lower value at 2.0 mW/cm^2^, while the lowest rate was observed in the case of 1.0 mW/cm^2^. For the samples, Au1 and Au2, the refractive index change has a maximum value of at 1.4 mW/cm^2^, while for the samples with a higher amount of gold nanoparticles, the refractive index changes in the same way as it does for the reference sample, i.e., it has a higher value at the higher intensity. The same process could be seen for the Ref sample, the refractive index change increased with the intensity change, and it will be largest for 2.0 mW/cm^2^.

### 3.2. Optical Transmission Spectroscopy Measurements and Transmission Electron Microscopy (TEM) Analysis

**Figure 2:**
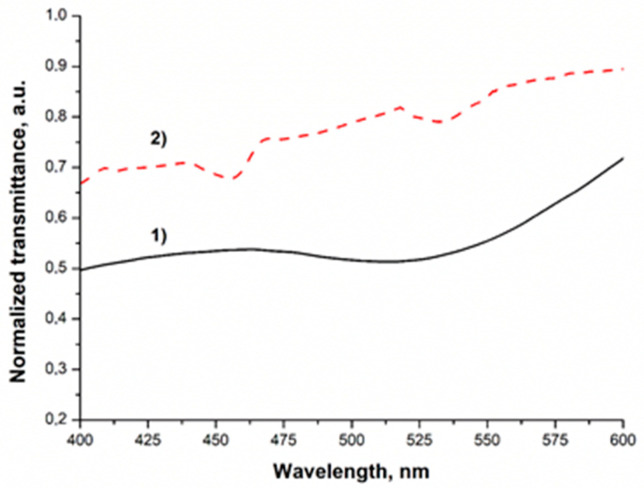
Normalized ultraviolet–visible (UV–VIS) transmittance spectra AuNP in toluene solution (1) and Au1 nanocomposite sample as a representative curve among the gold nanoparticles containing samples (2).

The transmission spectroscopy measurements can confirm the plasmonic effect of the AuNPs in the resin matrix. The TEM micrographs can give information about the distribution of AuNPs in polymerized resin. The nanocomposites created were measured with a spectrophotometer to show the suitability of using the gold nanoparticles to enhance the properties of the modified resin. The measured transmittance spectra of the investigated samples were normalized to show the characteristics of the spectra more clearly. The normalized UV–VIS transmittance spectra of the Au1 sample and AuNP in toluene are shown in Figure 2. The spectral feature of the green LED light source and the Ref samples were shown in our previous paper [16].

It was shown that the light source is operating in the region where the photoinitiator absorbs the light. Based on the results, it can be seen that this light source emits green light, which could excite the plasmon field of the added gold nanoparticles.

**Figure 3:**
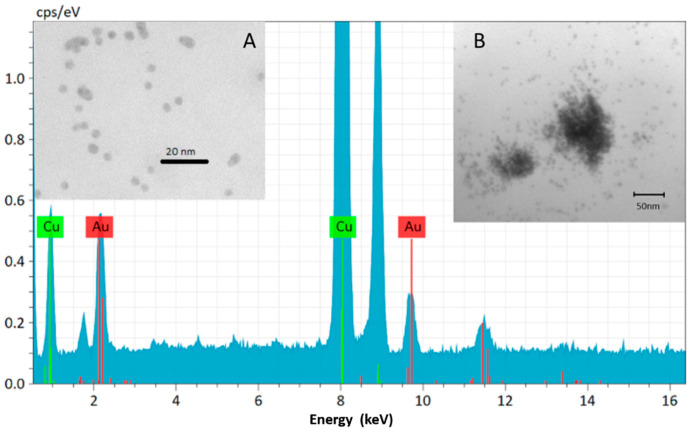
Representative transmission electron microscopy picture from gold nanoparticles (**A**,**B**) inside the cured resin matrix equipped with an X-ray elemental analysis spectrum.

It can be seen in Figure 2, that the first peak of sample 2, around 460 nm, originates from the photoinitiator [16]. According to our previous results, the LED light source could enhance the photopolymerization because it emits light in the region where the initiator absorbs the light [16]. It was also observed that the LED light source emits light in the same light region as the gold nanoparticles in toluene and the Au1 sample absorbs it, so the enhancement of the plasmon field can take place. The AuNP-doped cured polymer sample was studied with TEM to show that the nanoparticles are present in the matrix and to obtain information about the distribution of the AuNPs. Based on the electron micrographs it was found that the AuNPs are present in two forms in the matrix (see Figure 3). One of these forms is a nanocluster, where the nanoparticles are aggregated. The other form is composed of single nanoparticles distributed in the polymer matrix. The X-ray elemental analysis revealed unambiguously that the small particles and clusters were formed from the gold.

### 3.3. Diametral Tensile Strength (DTS) and Degree of Conversion (DC) Analysis

In the previous refractive index change investigations, our goal was to study whether we can create nanocomposites with gold nanoparticles and observe how the AuNPs of different concentrations influence the photopolymerization process. As shown, significant increases in the refractive indices were found for samples Au1 and Au2, indicating the most effective photo-polymerization reactions occurring in these samples. Consequently, we used these samples for further investigations.

**Figure 4:**
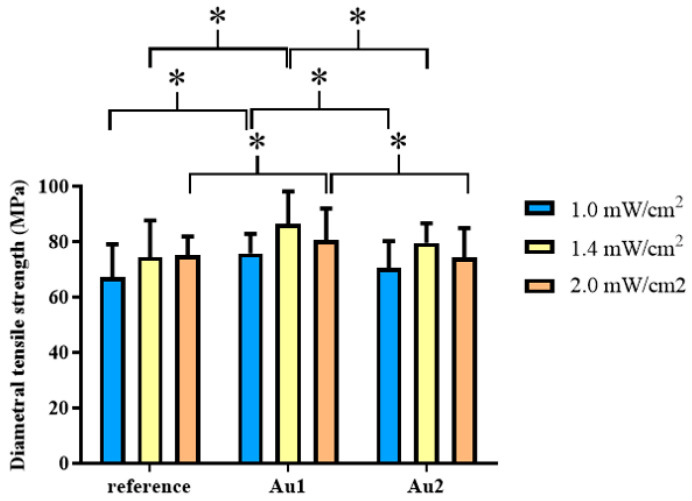
Mean values of diametral tensile Strength (DTS) of reference resin and a resin containing different concentrations of gold nanoparticles (Au1; Au2) at 1.0, 1.4, and 2.0 mW/cm^2^ light intensities. * *p* < 0.05.

The properties of the prepared samples were studied using different techniques. The DTS measurements give information on the cohesive strength of the polymer [20]. The diametrical acting compressive force results in a tensile force in the transverse direction inside the samples. Thus, for analyzing the influence of gold nanoparticles on the mechanical properties of the polymer nanocomposites, DTS values were determined for Ref and Au1 and Au2 nanocomposites. The results of DTS measurements are shown in Figure 4.

The DTS studies showed different trends for the reference sample and the samples with AuNPs as a function of the light intensity (Figure 4, Table 4, Table 5). The DTS values increase in the order of 1.0 mW/cm^2^ < 1.4 mW/cm^2^ < 2.0 mW/cm^2^ for the reference resin and 1.0 mW/cm^2^ < 1.4 mW/cm^2^ > 2.0 mW/cm^2^ for the Au1 and Au2 samples. A larger value was obtained for the Au1 (DTS 86.39 MPa) and Au2 (DTS 79.52MPa) samples cured at 1.4 mW/cm^2^ light intensity. The DTS data of AuNPs containing samples increased by the increase of light intensity, however, the differences were not significant (*p* > 0.05), similarly to the Ref sample. In the aspects of the presence of AuNPs, the tendencies were Ref < Au1 > Au2 at all intensities. Although sample Au2 showed higher DTS value compared to the Ref sample except for 2 mW/cm^2^, the differences were not significant. Moreover, sample Au1 showed significantly higher DTS value compared to Ref (*p* < 0.05) at all intensities. Among the measured data, the Au1 (AuNPs 0.0208 wt%) sample polymerized with 1.4 mW/cm^2^ light intensity showed the highest DTS (86.39 MPa) value. As expected, the reference resin produced higher DTS value with increasing intensity, because of the higher energy output and absorption capacity per cm^2^ inside the sample.

**Figure 5:**
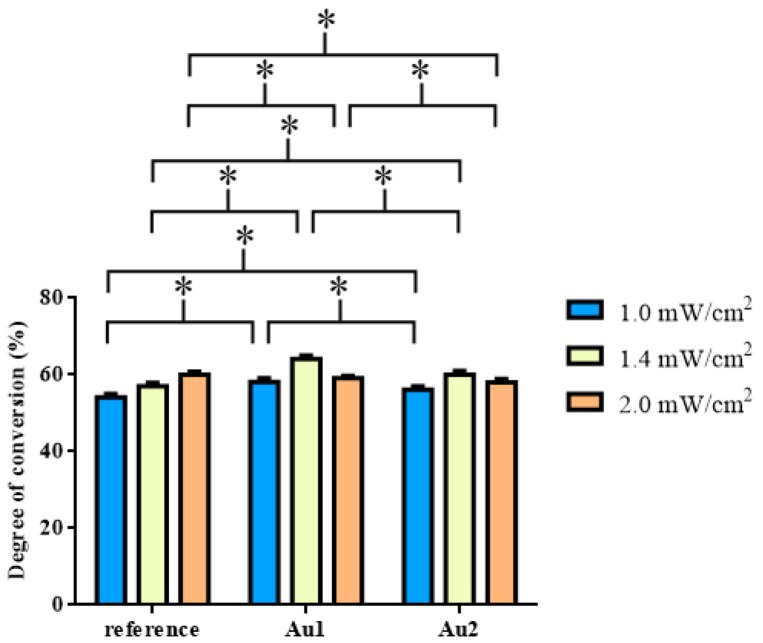
Mean values of degree of conversion (DC, %) of reference resin and a resin containing different concentrations of gold nanoparticles (Au1; Au2) at 1.0, 1.4, and 2.0 mW/cm^2^ light intensities.* *p* < 0.05.

The degrees of the conversion were also determined by Raman spectroscopy and the results of these measurements are shown in Figure 5.

The samples were measured before and after illumination at the surface. DC was different for the three samples (see Figure 5, Table 6, Table 7). The DC data showed a maximum at 1.4 mW/cm^2^ at AuNPs containing samples Au1 (DC 64.14%) and Au2 (DC 60.02%). For the reference resin, the highest DC (Ref DC 60.06%) value was obtained at 2 mW/cm^2^ light intensity, however, the differences between the compared pairs were not significant. Similar to the refractive index change and the DTS measurements, the Au1 composite has significantly higher DC for intensities of 1.0 mW/cm ^2^ (Au1 DC 58.07%) and 1.4 mW/cm^2^ (Au1 DC 64.14%) than the reference and Au2 samples. At 2.0 mW/cm^2^ the tendency changed: Ref (DC 60.06%) > Au1 (DC 59.11%) > Au2 (.DC 58.06%) The undoped reference resin showed significantly higher DC than samples Au1 and Au2. Among the measured DC data, the Au1 sample at 1.4 mW/cm^2^ showed the highest value following the results of DTS and SPRi measurements.

## 4. Discussion

This study aimed to analyze the effect of the thermo-plasmonic effect of AuNPs in an experimental dimethacrylate-based resin. It is well known that AuNPs display surface plasmon resonance (SPR) as a result of irradiating them at a targeted light frequency. SPR yielded a heat to increase the temperature of the surrounding environment and possibly enable the polymerization process. In this study, the AuNP-induced photopolymerization efficiency was investigated by SPRi, transmittance, diametral tensile strength, and degree of conversion. The SPRi measurements were used as a filter to find the optimal AuNPs concentration.

Generally, during a photopolymerization process, inefficient light transmission is a result of surface reflection, photoinitiator, and pigment/dye absorption, scattering by filler particles, and interfacial filler/resin refraction. As the polymerization reaction proceeds, the optical properties change, and the refractive index rises due to a rapid increase in cross-link density and viscosity. Consequently, the polymerization reaction can be monitored with the help of the change of the refractive index in time, thus it seemed to be a suitable method for examining the polymerization kinetics [21,22]. In our investigation, increase in the refractive index was detected at undoped reference resin (Table 1). The measured indices (1.466–1.494) are similar to those reported in the literature [23,24]. The changes of the refractive index in sample Ref, as a function of light intensity, corroborate well with our earlier work, in which we observed a significant light intensity dependence of the conversion of Irgacure 784-dimethacrylate resin [16]. The higher intensity of light was used, the higher refractive index change was detected.

It is visible that the addition of AuNPs to the reaction mixture influences the rate of the photo-polymerization, thanks to their thermoplasmonic effect, i.e., combined effect of temperature and plasmon field. Table 3 summarizes the change of the refractive index at different light intensities. Au1, Au2, samples showed the highest refractive index change at 1.4 mW/cm^2^, the lower value was found at 2.0 mW/cm^2^, and the lowest is at 1.0 mW/cm^2^ light intensities. We have not observed a significant difference between the refractive index changes. Another important consequence of this measurement is that the Au1, Au2, samples with 0.0208 wt%, 0.0416 wt% AuNPs concentration, respectively, showed the best properties among the samples investigated. Others have found that independently of the composition of the mixture, the refractive index of a photopolymerizable, undoped acrylic formulation varied linearly with the conversion during the reaction. It was also emphasized that the refractive index value of a photopolymerizable medium only depended on the conversion and the temperature, as reported for the Bis-GMA/TEGDMA unloaded resins with a blue light-sensitive photoinitiator system [22]. Govorov et al. have published a theoretical study in which they estimated the typical time to significantly increase the temperature of the surrounding material (water, ice, and polymer) using a single AuNP and a collection of AuNPs, and described the effect of collective plasmon resonance for the heating enhancement [25]. They concluded that the light-excited AuNP with light could increase temperature and even melt the surrounding medium. The collective applied AuNPs superstructure can act as an amplifier of the heating effect and also create local areas of high temperature, hot spot (collective plasmon resonance). Because of this, adding AuNPs to the dimethacrylate resin could work also as a hot spot and heat amplifier in our experimental resin to reach the higher conversion. When Au NPs are considered in one medium, this particle is reactive and generates heat, electrons, electric fields and scatters light, on the one hand, it is considered as a solid particle acting as an obstacle in the direction of the exciting light, on the other hand. At higher concentrations, the high number of metal particles and the additional agglomeration of the particles (creating big clusters/obstacles in the direction of the light) can work as an “optical inhibitor” of the photo-polymerization. Thus, if the AuNPs concentration is higher as in the case of samples Au4, Au5 and Au6, such optical inhibition is present, yielding longer initiation time and lower refractive index increment. From the other point of view higher refractive index change was observed for Au1 (0.0326), Au2 (0.0304), at 1.4 mW/cm^2^ than at 2.0 mW/cm^2^ light intensity. It seems that the optimal intensity of light is around 1.4 mW/cm^2^. This phenomenon may be accounted for by the fact that at 2.0 mW/cm^2^ light intensity extensive formation of primary radicals from the initiator can take place, which leads to a rapid polymerization resulting in the formation of an incomplete network. The incomplete network probably has a lower refraction index and index increment. In addition, as the polymerization of dimethacrylates proceeds, crosslinked network forms and the propagation becomes diffusion-controlled causing a significant decrease of the polymerization rate (Rp) [3]. Other researchers have tested embedded silver nanoparticles (AgNPs) in epoxy and methacrylate resins. They have detected a marked increase in the temperature in the extent of polymerization. They have also stated that the principle of plasmonic heating of AgNPs under 420 nm light irradiation can be used to perform the polymerization of a dimethacrylate-based resin initiated by benzoyl peroxide in the absence of photoinitiator. The heat released by the AgNPs results in the thermal decomposition of the benzoyl peroxide and initiates the polymerization [14]. The possible explanation of our results could be that the light excitation of AuNPs in the resin results in a temperature increase with a help of the thermo plasmonic effect, and the elevated temperature kinetically accelerates and increases the rate of the photo-polymerization procedure. At the same time the presence of a direct plasmon field effect on electron transitions, chemical bonds transformations may be also supposed.

When a metal nanoparticle is illuminated, the intercepted light is partly scattered in the surroundings, and the other part is absorbed and finally dissipated into heat. The balance between scattering and absorption is substantially size-dependent. For instance, while small gold spheres smaller than 10 nm in diameter mainly act as invisible nano sources of heat, scattering processes dominate for diameters larger than 50 nm [26]. Our dodecanethiol-functionalized spherical gold nanoparticle size is 5 nm, can be potentially able to increase the temperature of the surroundings. The transmittance curves (Figure 2) demonstrate the absorption of the light in the 525–550 nm spectra, as can be seen on the curves of AuNPs in toluene solution, and AuNPs containing the nanocomposite. The peak at around 450 nm represents the Irgacure 784 photoinitiator transmittance. Earlier Trujillo et al. demonstrated the significant influence of temperature rising on the polymerization rate and conversion of dental composites [11]. When increasing the temperature of dimethacrylate-based dental composite within the potential biologically compatible limit, increasing polymerization rate and degree of conversion was observed.

According to the literature, statistically, dental composites displayed sufficiently brittle behavior for the diametral tensile test (DTS) to be valid for evaluation of the tensile strength of newer dental composites [27]. The influence of UDMA, Bis-GMA, and TEGDMA on selected mechanical properties was investigated by Asmussen and Peutzfeldt [2]. With respect to the fact that these monomers have different molecular stereochemistry and influence on mechanical properties may be different, the mixing ratio is determined by the intended mechanical property of the composite. They observed that DTS increased when Bis-GMA or TEGDMA is replaced by UDMA and when Bis-GMA is replaced by TEGDMA. They explained these findings by the degree of conversion of the polymer matrix and referred to their earlier publication in which they realized the dependence of DTS on the degree of conversion (DC) of the methacrylate double bonds. In their discussion, they concluded that the different monomers could behave differently. Flexible monomer molecules or the ability of urethane linkage to form hydrogen bonds in the copolymer presumably results in restricted sliding of the polymer segments relative to each other [2,28]. The DTS data of our reference resin follow the measurements of Asmussen and Peutzfeldt. They tested experimental composites with different ratios of common dimethacrylate (TEGDMA, UDMA, Bis-GMA) components. Their measured mechanical parameters were lower, but they tested (silanized glass) filled resins. Barszczewska-Rybarek has published several factors (chemical structure of dimethacrylate molecules and the formed copolymer network, crosslink density, the degree of conversion) that affect the mechanical properties of the forming polymer [29]. The literature data prove that the DC is the most evident parameter, defining the dimethacrylate polymer network structure. This is also the most often used technique when structure–property relationships are being investigated. DC highly depends on the monomer chemical structure, initiation technique, curing time, sample thickness, initiator systems, and its concentration, irradiation time and source, and filler content. The minimum DC in the case of conventional dental composites is between 50–55%. The lower DC parameters result in unacceptable clinical use. Homopolymers were arranged according to the following order by limiting DC: Bis-GMA < Bis-EMA < UDMA < TEGDMA. Crosslink density is also an important factor from the point of view of mechanical properties. More theoretical models try to describe the relation of different factors (molecular weight, degree of conversion, double bond concentration). The physical crosslinking in dental dimethacrylate polymer networks results from hydrogen bonding. Hydrogen bonding determines the dimethacrylate monomer viscosity. The lower the viscosity of the dimethacrylate mixture, the higher the degree of conversion. Several instrumental methods are available to allow the DC determination in dimethacrylate polymer networks. Infrared spectroscopy: Fourier-Transform Infrared Spectroscopy (FTIR), Attenuated Total Reflection FTIR, (ATR-FTIR), Near-Infrared Spectroscopy (NIR); Raman spectroscopy, Differential Scanning Calorimetry (DSC) and solid-state Nuclear Magnetic Resonance (ssNMR) are particularly readily used.

Our dimethacrylate resin contained UDMA (53,3%), TEGDMA (25,4%), and Bis-GMA (21,4%) and, therefore, this resin is rich in hydrogen bonds and suitable for cross-link formation. Given the length and elasticity of different dimethacrylate monomers, the theoretical and real crosslink density of their copolymer network affects indirectly the mechanical parameters. The high viscosity aromatic rigid Bis-GMA molecule limits the DC. TEGDMA exhibits relatively high DC, because of favorable stereochemistry. The long flexible chain of dimethacryate glycol acts as a diluent. UDMA is considered also a viscosity reducer and increases DC. The flexibility of urethane linkage why adding this molecule to the mixture to provide better toughness. Our DC data follow the literature. Dental composite displays DC data in the range of 50–77% [30,31,32]. The highest DC and DTS values were measured in the case of the Au1 sample at 1.4 mW/cm^2^. Therefore, the ideal AuNPs concentration was applied in sample Au1. Related to the reference resin the higher DC and the consequent higher DTS data could be explained with the presence of AuNPs, and their plasmon effect on the polymerization. From the other side, the lower Au2 DTS (78,92 MPa) and DC (60%) related to Au1 DTS (82,86 MPa) and DC (64%) can be explained with the increased optical inhibition effect of higher AuNPs content.

If we relate the data (DTS, DC) at 1,4 mW/cm^2^ and 2.0 mW/cm^2^ it is clear that we obtain lower data at higher intensity whereas differences are not significant. In our earlier work, we described this new dimethacrylate resin containing Irgacure 784 photo-initiator. We showed that the cross-link density does not necessarily change if we increase the light intensity, showing that Irgacure 784 could work successfully at narrow intensities which means the intensity of light is probably not a determinant factor for the results.

Isolated and cluster-forming AuNPs could be seen on the TEM image. Nanoparticles represent the high surface area and display a tendency to agglomerate and form clusters. In the literature, numerical modeling of the temperature evolution time and space was found in the system which contains differently arranged AuNPs. This modeling showed the more agglomerated the AuNPs are, the higher the temperature near that area and the longer the time to reach thermal equilibrium. Thus agglomeration is not ideal for steady heat distribution, but cannot inhibit it [33]. It seems that AuNPs are applicable to develop and improve dental composite resin, but further investigations are needed.

The limitation of our work is that our material does not contain inorganic filler particles. We applied special initiator (Irgacure 784) that has not been applied in dental resin yet. For the photo-activation we used an experimental LED curing unit that emits in the green light spectra. Our functionalized AuNPs are in a diameter of 5 nm that is not the usual size applied in combination with experimental resins in the literature. We applied low light intensities for the initiation, which is not common in dentistry. In vitro tests have not been undertaken in connection with our experimental resin yet.

## 5. Conclusions

In our experiment, we successfully incorporated dodecanthiol functionalized 5 nm-sized AuNPs in dental dimethacrylate resin containing Irgacure 784 photoinitiator.

The SPRi measurements of refractive index change and Raman microscopy/confocal Raman spectroscopy enabled us to determine the degree of conversion and to monitor the progress of the polymerization reaction in the resin.

Diametral tensile stress and degree of conversion data were improved related to the reference resin.

We were able to find the optimal light intensity (1.4 mW/cm^2^) and gold concentration Au1 (0.0208 wt%).

The clinical importance of our work is that a new dimethacrylate-based experimental resin was produced which possesses better physical and chemical properties than the reference resin, and can be applied as a resin matrix of an experimental dental composite.

## Figures and Tables

**Table 1:** Gold nanoparticles content (wt %) in different composites.

Sample Code	Au NPs Content (wt %)
Reference (Ref)	-
Au1	0.0208
Au2	0.0416
Au3	0.0833
Au4	0.1665
Au5	0.3330
Au6	0.6660

**Table 2:** The refractive index measurements and the calculated absolute refractive index change of the reference sample at 1.0, 1.4, and 2.0 mW/cm^2^ light intensities after 2 min irradiation.

Light Intensity (mW/cm^2^)	Before Illumination	After Illumination	Absolute Change
1.0 mW/cm^2^	1.466 ± 0.002	1.483 ± 0.002	0.017 ± 0.002
1.4 mW/cm^2^	1.466 ± 0.002	1.490 ± 0.002	0.024 ± 0.002
2.0 mW/cm^2^	1.466 ± 0.002	1.494 ± 0.002	0.028 ± 0.002

**Table 3:** The refractive index change of the investigated samples at 1.0, 1.4, and 2.0 mW/cm^2^ light intensities measured by ellipsometry.

Samples	1.0 mW/cm^2^	1.4 mW/cm^2^	2.0 mW/cm^2^
Ref	0.0155	0.0250	0.0277
Au1	0.0202	0.0326	0.0309
Au2	0.0182	0.0304	0.0296
Au3	0.0163	0.0228	0.0255
Au4	0.0143	0.0177	0.0250
Au5	0.0133	0.0163	0.0224
Au6	0.0120	0.0138	0.0218

**Table 4:** Statistical analysis of Diametral Tensile Strength data (mean, SD values).

Intensities	Samples	Mean (DTS, MPa)	SD
1.0 mW/cm^2^	reference	67.171	11.884
	Au1	75.811	6.951
	Au2	70.554	9.688
1.4 mW/cm^2^	reference	74.464	13.198
	Au1	86.392	11.726
	Au2	79.524	7.068
2.0 mW/cm^2^	reference	75.112	6.779
	Au1	80.454	11.491
	Au2	74.389	10.508

**Table 5:** Statistical analysis of diametral tensile strength data (*p* values).

Intensities	Samples	*p* Value
1.0 mW/cm^2^	ref-Au1	0.00013
	ref-Au2	0.06686
	Au1-Au2	0.00611
1.4 mW/cm^2^	ref-Au1	0.03186
	ref-Au2	0.48792
	Au1-Au2	0.04567
2.0 mW/cm^2^	ref-Au1	0.00023
	ref-Au2	0.06044
	Au1-Au2	0.04695

**Table 6:** Statistical analysis of degree of conversion (DC) data (mean, SD values).

Intensities	Samples	mean (DC, %)	SD
1.0 mW/cm^2^	reference	54.098	0.923
	Au1	58.071	1.014
	Au2	56.154	0.906
1.4 mW/cm^2^	reference	57.030	0.923
	Au1	64.137	0.872
	Au2	60.020	1.003
2.0 mW/cm^2^	reference	60.056	0.790
	Au1	59.113	0.683
	Au2	58.057	0.924

**Table 7:** Statistical analysis of degree of conversion data (*p* values).

Intensities	Samples	*p* Value
1.0 mW/cm^2^	ref-Au1	4.6 × 10^−14^
	ref-Au2	9.4 × 10^−8^
	Au1-Au2	9.1 × 10^−7^
1.4 mW/cm^2^	ref-Au1	9.5 ×10^−23^
	ref-Au2	7.1 × 10^−11^
	Au1-Au2	7.1 × 10^−15^
2.0 mW/cm^2^	ref-Au1	0.00053
	ref-Au2	4.8 × 10^−8^
	Au1-Au2	0.00043
